# Surgical Management of Discoid Lateral Meniscus With Anterior Peripheral Instability: Retaining an Adequate Residual Meniscus Volume

**DOI:** 10.1016/j.eats.2022.02.021

**Published:** 2022-06-08

**Authors:** Yusuke Hashimoto, Shinya Yamasaki, Dan Guttmann, John B. Reid, Sean Marvil, Takuya Kinoshita, Hiroaki Nakamura

**Affiliations:** a Department of Orthopaedic Surgery, Osaka City University Graduate School of Medicine, Osaka, Japan; b Department of Orthopaedic Surgery, Osaka City General Hospital, Osaka, Japan; c Taos Orthopaedic Institute, Taos, New Mexico, U.S.A

## Abstract

Discoid lateral meniscus (DLM) presents with differing pathoanatomy and may exhibit various types of tears. The treatment strategy is based on the presence and location of instability as a result of deficient capsular attachment. Recently, meniscal stabilization after saucerization has been recommended for DLM to preserve the meniscus shape, prevent extrusion, and mitigate against the progression of osteoarthritis. In addition to stabilization, the resection volume is important to prevent osteoarthritic changes. Although there was no tear and no displacement of the lateral meniscus on magnetic resonance imaging, some DLMs were found to have tears and peripheral instability during arthroscopy. Therefore, the assessment of peripheral instability during surgery is very important to achieve a desirable clinical outcome. This Technical Note describes an arthroscopic technique for anterior peripheral stabilization of the DLM, in which we highlight the surgical procedure for repair of the anterior horn, reassess the instability around the popliteal hiatus after the anterior horn is repaired, and the stabilization of the posterior horn, if necessary.

Discoid lateral meniscus (DLM) is a congenital anatomic variation in meniscal shape and stability, rendering the lateral meniscus susceptible to injuries and instability that often require surgical management. Previously recommended treatments for symptomatic DLMs were subtotal or total meniscectomy. This was because of the high incidence of tears and degenerative lesions associated with DLM. Current treatment algorithms emphasize meniscal rim preservation with arthroscopic saucerization. With recent arthroscopic advancements, for patients with unstable peripheral rims, saucerization with repair has been advocated recently to prevent extrusion in an attempt to minimize the associated risk of degenerative arthrosis.^1^

Anterior peripheral instability, including the posterocentral type of DLM, identified with magnetic resonance imaging (MRI), is sometimes associated with a snapping phenomenon and instability of the anterior horn of the meniscus,^2^ as opposed to the no-shift type of DLM on MRI, hiatus widening,^3^ suggesting meniscal instability in the nondiscoid meniscus. Bulging of the meniscal margin^4^ and absence of the popliteomeniscal fascicle may indicate meniscal instability of the DLM.^5^ This Technical Note describes an arthroscopic technique designed to address anterior and peripheral stabilization of the DLM. We highlight the surgical repair of the anterior horn of the DLM and reassess the instability around the hiatus following repair of the anterior horn. We also describe the addition of a suture to address the posterior horn of the DLM in case of posterior instability after anterior repair.

## Surgical Technique (With Video Illustration)

**Fig 1:**
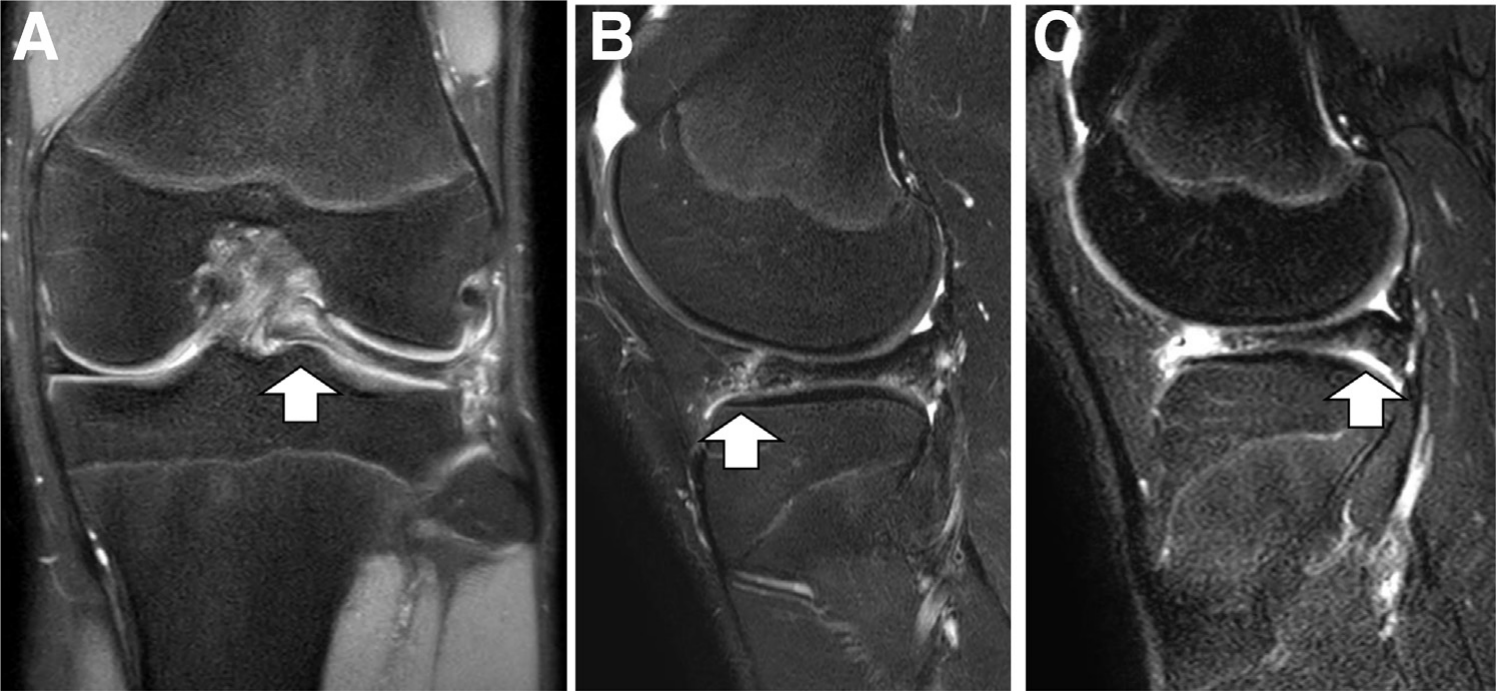
(A) Coronal, (B) sagittal, and (C) sagittal with full-extension knee magnetic resonance images of anterior instability of the discoid lateral meniscus (DLM) in the left knee. The DLM was displaced posterocentrally on the coronal image (A; white arrow), and anterior parameniscal soft-tissue edema was seen on the sagittal image (B; white arrow). The DLM is displaced posteriorly, and the posterior horn appears to be thick on the sagittal images with knee full extension (C; white arrow).

This technique is indicated for cases in which the periphery of the anterior horn has become detached from the capsule and the entire DLM has been displaced from the normal anatomic position to one that is posteriorly or posterocentrally displaced (Fig 1A). This pathoanatomy often has a snapping phenomenon, anterior parameniscal soft-tissue edema, and bulging of the meniscal margin frequently observed on MRI^4^ (Fig 1B). Furthermore, MRI can demonstrate a posterior shift of the meniscus with the knee in full extension (Fig 1C).^2^^,^^6^

**Fig 2:**
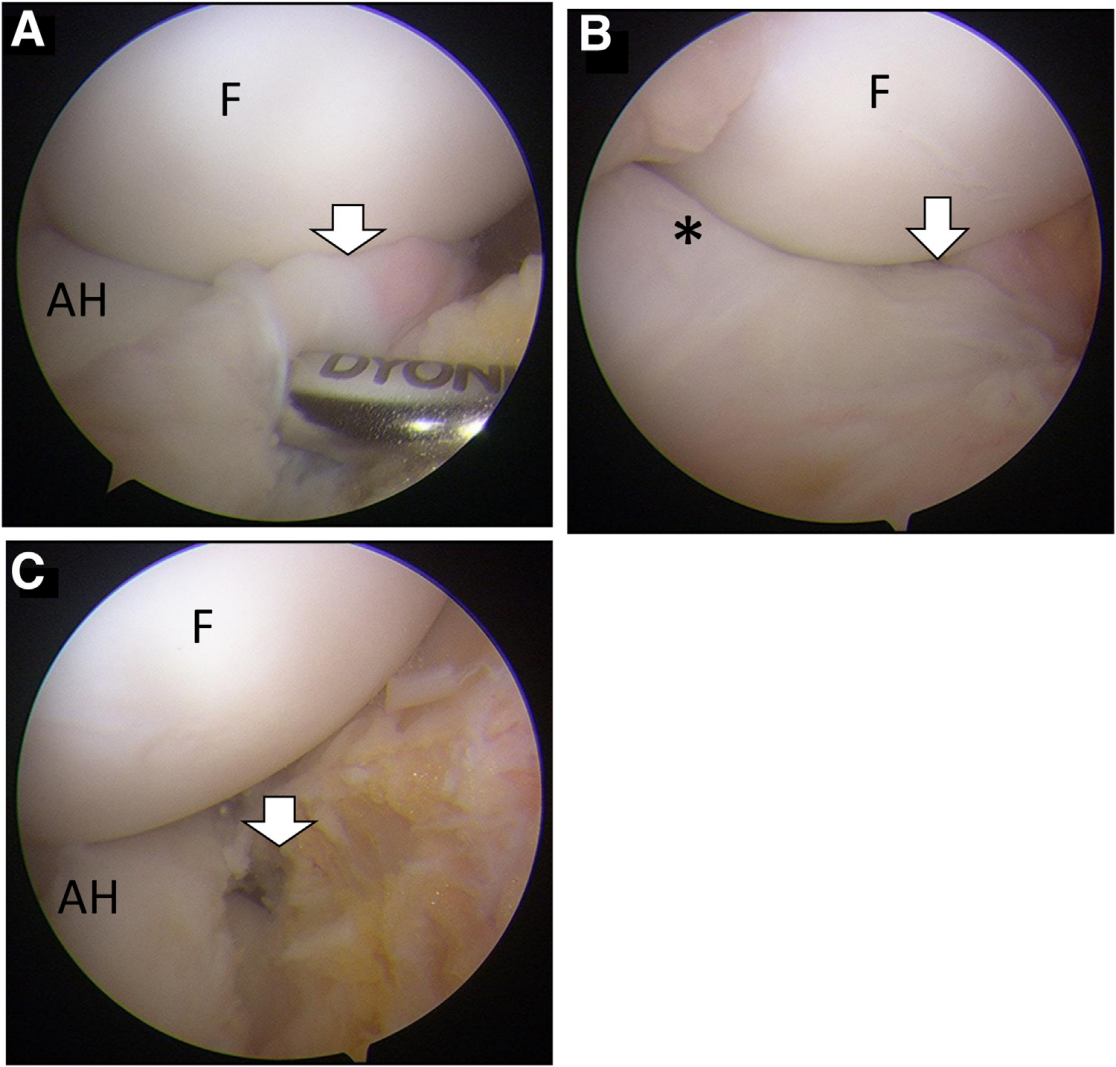
Arthroscopic findings of the left knee viewed from the anteromedial portal in the supine position. (A) Connective tissue (white arrow) between the anterior horn of DLM and capsule is found with arthroscopic viewing from the anteromedial portal in the flexed knee position. (B) The anterior horn of DLM (black asterisk) is moved posteriorly under knee extension. The connective tissue (white arrow) still remains between the DLM and capsule (white arrow). (C) The detached periphery of the anterior horn is found after removal of the connective tissue (white arrow). (AH, anterior horn of DLM; DLM, discoid lateral meniscus; F, femoral condyle.)

**Fig 3:**
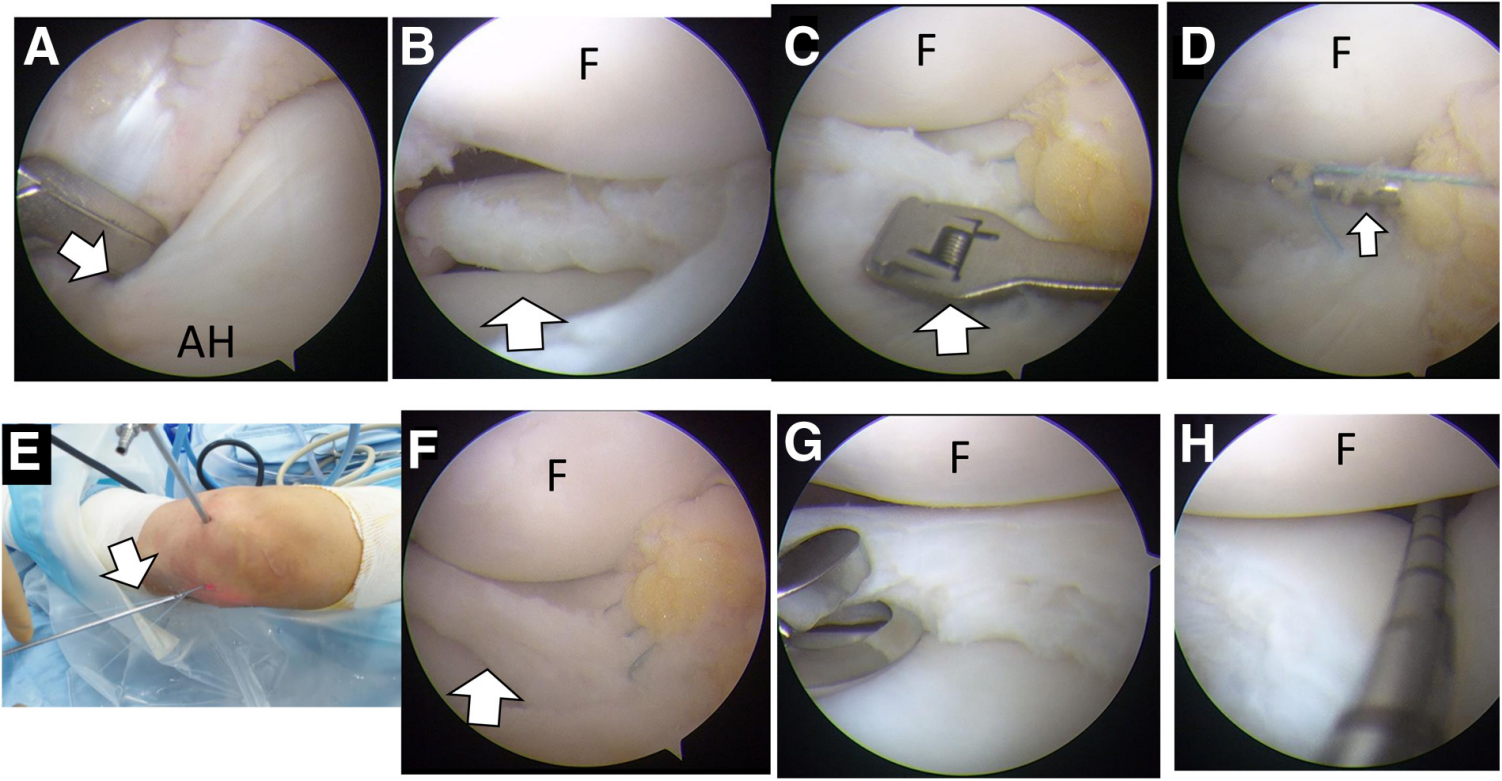
Saucerization of the lateral meniscus in a left knee, viewed from the anterolateral portal in the figure-four position. (A) Saucerization is started from the border between the anterior horn and central area (white arrow) of the DLM with a 45° punch from the anteromedial portal parallel to the circumferential fibers of the anterior horn of the DLM viewing from anterolateral portal. (B) Saucerization with removal of the central area (white arrow) is performed with punch from the anteromedial portal. (C) Sutures with 2-0 FiberWire are then passed through the anterior horn using a Scorpion suture passer (white arrow) introduced through the anterolateral portal. (d) A NanoPass (Stryker, Kalamazoo, MI) (white arrow) is then introduced through the anterolateral portal and used to penetrates the upper side of capsule and reach femoral side of DLM and retrieve the upper side of suture. (E) A suture is tied using a sliding knot technique and secured with a knot pusher (white arrow) through the anterolateral portal. (F) After stabilization of the anterior meniscus, the residual posterior meniscus is displaced anteriorly and appears larger (white arrow). (G) After the residual meniscus of posterior horn is remeasured, the posterior horn is resected until 10 mm from the hiatus is preserved again. (H) Meniscal instability is again evaluated with a probe. (AH, anterior horn of DLM; DLM, discoid lateral meniscus; F, femoral condyle.)

With the patient in the supine position, a nonsterile tourniquet is applied to the upper thigh of the operated leg. Arthroscopic evaluation is performed using standard anterolateral and anteromedial portals. DLM is diagnosed from the anterolateral portal view; no widening of the hiatus and abnormal movement of the DLM under extension and deep flexion of the knee (Video 1) is confirmed from the lateral gutter view through the anterolateral portal. From the anteromedial portal view, the periphery of the anterior horn is sometimes not detached from the capsule (Fig 2 A and B), similar to a ramp lesion^7^; however, the entire DLM is displaced posteriorly from flexion (Fig 2A) to extension (Fig 2B) of the knee (Video 1). Connective tissue is easily removed with an arthroscopic shaver, and the site of the tear is demonstrated (Fig 2C). Saucerization is performed from the transition between the anterior horn and central area of the DLM using a 45° punch parallel to the circumferential fibers (Fig 3A).^8^ After measuring a 1-cm resection length (Video 1) with a depth gauge (TRUKOR Depth Gauge; Smith & Nephew, Andover, MA), the posterocentral portion is then resected until reaching a point 10 mm from the hiatus (Fig 3B). A 2-0 FiberWire suture (Arthrex, Naples, FL) is then passed through the anterior horn using a Scorpion suture passer (Arthrex) introduced through the anterolateral portal (Fig 3C). A NanoPass (Stryker, Kalamazoo, MI) is then introduced through the anterolateral portal and used to penetrate the lower side of the capsule and tibial side of the DLM and retrieve the lower limb of the suture. Next, the same procedure is performed to penetrate the upper side of the capsule to reach the femoral side of the DLM and retrieve the upper limb of the suture (Fig 3D). The suture is tied using a sliding knot technique and secured with a knot pusher (Fig 3E); this technique is then repeated. The total number of sutures used is determined based on the length of the tear. The distance between sutures is 3 mm (Video 1).

**Fig 4:**
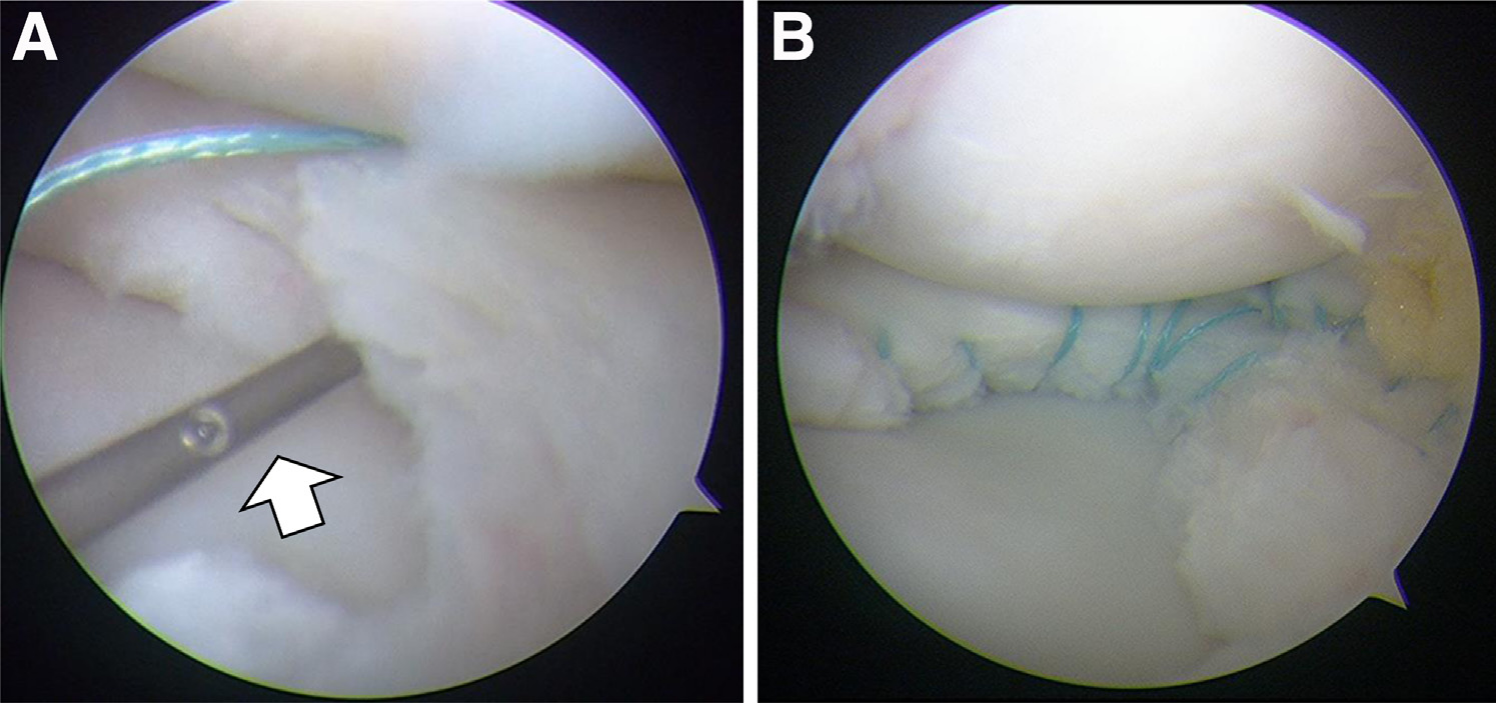
Additional inside-out suture of the left knee viewed from the anterolateral portal in the figure-four position. (A) The dual meniscal repair needles loaded with 2-0 braided polyester sutures (white arrow) penetrate the unstable portion of the meniscus through a cannula positioned in the anteromedial portal. (B) Arthroscopic view after saucerization with inside-out repair from the anteromedial portal.

After stabilization of the anterior meniscus, the residual posterior meniscus is often displaced anteriorly and appears larger (Fig 3F, Video 1). After resection, the length is remeasured, and the posterior portion of the meniscus is resected until 10 mm from the hiatus is preserved (Fig 3G, Video 1). Meniscal instability is again evaluated with a probe (Fig 3H); if instability persists in the posterior horn, surgical stabilization is performed. The skin incision is parallel to and just posterior to the lateral collateral ligament with the knee in 90° of flexion for a standard inside-out meniscal repair. The fascia is exposed and cut immediately posterior to the lateral collateral ligament. A retractor (Stryker) is inserted into the interval between the lateral posterior capsule and the gastrocnemius to protect the neurovascular structures behind the knee. Once the retractor is in place, meniscal repair is performed through the anteromedial portal in the figure-of-four-leg lock position. After preparing the repair site with the arthroscopic rasp, dual meniscal needles loaded with 2-0 braided polyester sutures (Stryker) are used to penetrate the unstable portion of the meniscus (Fig 4A, Video 1) using a cannula (Stryker) positioned in the anteromedial portal. The suture needles are retrieved under direct visualization through a previously prepared lateral incision. The sutures are tied over the capsule. The technique is performed with stitches placed at 3-mm intervals using 2-0 nonabsorbable sutures. After repairing the posterior portion, the width of the repaired meniscus and stability of the meniscus are confirmed (Fig 4B, Video 1).

**Fig 5:**
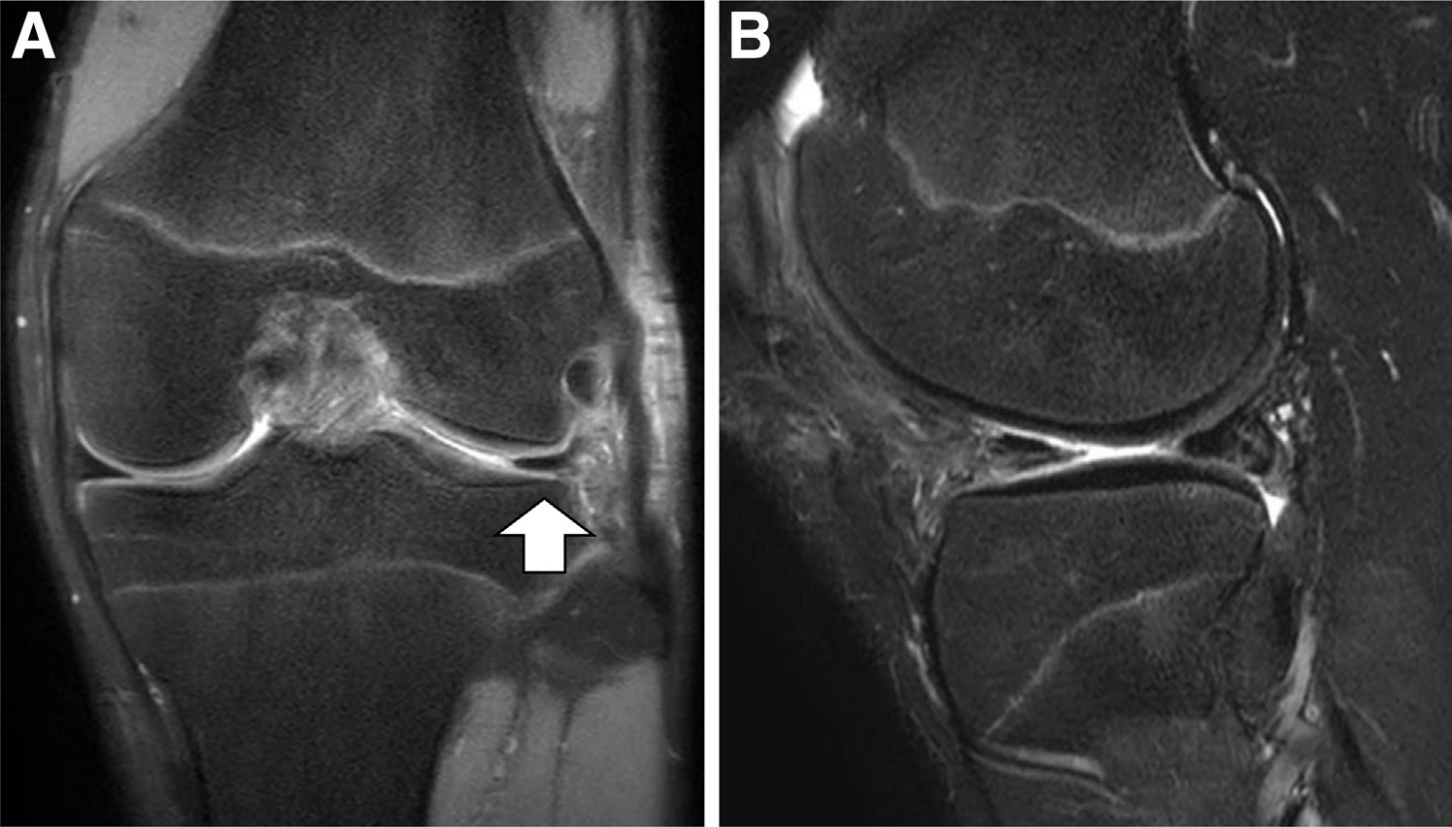
Postoperative magnetic resonance imaging after saucerization with the transportal transcapsular repair for anterior instability and additional inside-out suture for consecutive posterior instability in the left knee. It resembled the normal meniscus. The width of the body of the lateral meniscus was 10 mm (A; white arrow).

The postoperative protocol for the patients in this study included immobilization with a brace for a week, limited knee range of motion of 0 to 90° for 3 weeks, and protected weight-bearing for 6 weeks. Patients were permitted to jog at 3 months after surgery and return to previous sports at 6 months after surgery. Postoperative MRI showed that the width of the body of the lateral meniscus was 10 mm on the coronal image and with no hiatus widening on the sagittal image, which was similar to the width of the normal lateral meniscus (Fig 5A and B). This suggests that this method can restore normal meniscal morphology and stabilization after saucerization with repair.

## Discussion

A high prevalence of symptomatic DLMs is associated with peripheral instability.^5^ The current treatment algorithm for symptomatic DLMs includes meniscal rim preservation and peripheral meniscal stabilization. Studies have shown that saucerization with suture stabilization of the peripheral meniscal rim results in fewer degenerative changes compared with total or subtotal meniscectomy.^7^ Although a 6- to 8-mm width of the remaining meniscus rims was recommended as an appropriate value in a previous study,^9^ the long-term result of saucerization has been shown to be associated with the progression of lateral compartment arthritis in 68.5% of patients.^9^ Moreover, Nishino et al.^10^ reported that the width of the anterior horn, midbody, and posterior horn decreased significantly from 3 to 24 months after surgery. Therefore, we feel that a larger residual meniscus width is required.

In addressing anterior instability, the outside-in and all-inside suture techniques have previously been reported in non-DLM knees. However, saucerization with repair of anterior instability of the DLM has rarely been reported. The proposed advantages our described technique are as follows: (1) It allows for accurate saucerization by defining the starting point and leaving a meniscal width of 10 mm; (2) requires only one portal for arthroscopic suturing; (3) no subcutaneous knot irritation associated with the anterior repair; (4) includes a strategy to reassess the stability of the posterior meniscus after repairing the anterior horn of the DLM and adding the suture for the posterior portion; and (5) this technique is technically straightforward, as it requires only standard meniscectomy and meniscal repair skills (Table 1).

**Table 1:** Advantages and Limitations of the Procedure

Advantages	Limitations
• Accurate saucerization by defining the starting point and the length of the cutting width of 1 cm. • Need only 1 portal to suture for anterior instability. • No subcutaneous knot irritation for anterior repair. • Applicable for horizontal tears. • Can assess the instability of the posterior part after repairing the anterior horn of DLM and add the suture for posterior part. • Need only standard meniscectomy and repair skills.	• Need specific meniscal suture instrumentation. • Need to reassess the length of residual meniscus and posterior instability after anterior repair. • Possibility of suture failure. • Possibility of reinjury after meniscal stabilization.

DLM, discoid lateral meniscus.

**Fig 6:**
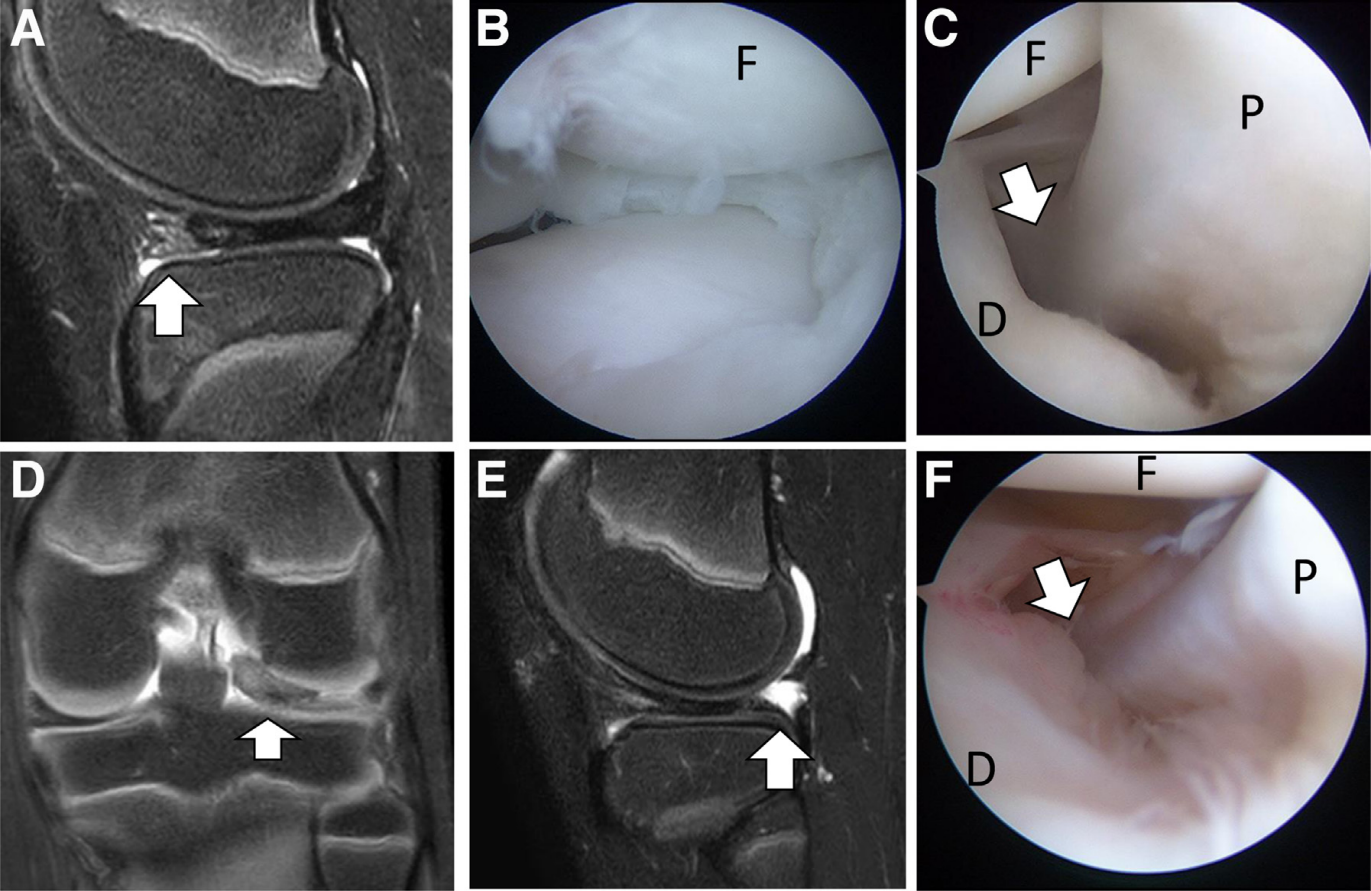
Newly developed case of posterior instability after anterior suture only for anterior instability of DLM in the left knee. (A) Anterior parameniscal soft-tissue edema (white arrow) was seen on the sagittal MRI. (B) Arthroscopic view after saucerization with transportal transcapsular repair for anterior instability from the anterolateral portal. (C) No hiatus widening (white arrow) was seen during first surgery. (D) The DLM is displaced anterocentrally (white arrow) on the coronal image after first surgery. (E) Meniscal loss of the posterior horn around the hiatus (white arrow) was seen on the sagittal images after first surgery. (F) Hiatus widening (white arrow) was seen in the revision surgery from the anterolateral gutter view. (D, discoid lateral meniscus; F, femoral condyle; MRI, magnetic resonance imaging; P, popliteal tendon.)

Regarding the evaluation of peripheral meniscal stability, bulging of the meniscal margin^4^, anterior parameniscal soft-tissue edema, and absence of the popliteomeniscal fascicle may indicate meniscal instability in DLM.^5^ Li et al.^3^ reported that widening of the popliteal hiatus, which can be seen on MRI, may lead to recurrent subluxation of the non-DLM. Meniscal instability sometimes develops due to hiatus widening after anterior repair (Fig 6A-F). Thus, reassessment for posterior instability is needed following anterior repair during surgery. If there is persistent instability of the posterior meniscus, then posterior repair is indicated. Regarding the suture technique, conventional outside-in suture repair techniques for anterior horn tears of the lateral meniscus have been reported to yield satisfactory results in clinical outcome studies.^11^ However, these techniques require an additional incision of approximately 1 to 2 cm or more in length, and subcutaneous knot irritation may also occur. All-inside repair techniques have been reported to avoid this skin incision and restore the normal biomechanics of the lateral meniscus during motion.^12^

This technique includes a combination of outside-in suture repair and all-inside repair techniques, which uses transportal transcapsular repair using specific meniscal repair devices. A possible limitation of this technique is that it requires specific meniscal repair devices and can is vulnerable to the risk of suture failure. Finally, as a technique paper, we cannot draw clinical conclusions; further follow-up is necessary to investigate the long-term results of this transportal transcapsular repair on clinical and radiologic outcomes.

The advantages and limitations, as well as the pearls and pitfalls, are summarized in Table 1, Table 2, respectively.

**Table 2:** Pearls and Pitfalls of the Procedure

Pearls	Pitfalls
• Careful evaluation of meniscal stability should be performed from the anteromedial and lateral gutter views through range of motion including extension and deep flexion of the knee. • A curved narrow basket punch rather than a straight punch is recommended to remove the anterior margin of the DLM. • Careful central debridement will preserve tissue to establish normal morphology. • The suture with Scorpion should be used to penetrate the unstable portion of the meniscus, including the horizontal tear. • NanoPass used to penetrate the upper side of capsule and retrieve the femoral side of suture. Next, the lower side of capsule is penetrated and the tibia side of suture is retrieved through the anterolateral portal. • Reassess the stability of posterior meniscus after repairing the anterior horn of DLM and add the suture for posterior stabilization if necessary.	• Anterior detachment of the meniscus is sometimes covered by a connective tissue–like ramp lesion of the medial meniscus. • Excessive debridement of either the DLM or capsule can eliminate the ability to create a normal meniscus shape. • Inadequate chondral clearance for suture passing may result in iatrogenic chondral injury. • After restoring anterior instability, checking the length of residual meniscus and the stability of the peripheral rim of the posterior meniscus are necessary. • Careful retraction should be performed to protect the neurovascular structures in the posterolateral aspect of the knee.

DLM, discoid lateral meniscus.

## Supplementary Data

Full ICMJE author disclosure forms

Video 1 In this video, we demonstrate the surgical management for anterior instability of the discoid lateral meniscus (DLM) in which we highlight the surgical procedure to repair the anterior horn of the DLM, reassess the stability around the hiatus after the anterior horn is repaired, and the posterior horn of the DLM is surgically stabilized if necessary. This case involved the left knee of a 16-year-old boy who had anterior instability of his DLM. The DLM was confirmed by arthroscopic inspection from the anterolateral portal in the figure-four position. No widening of the hiatus was observed, and abnormal movement of the DLM from the flexion to extension position of the knee was confirmed from the lateral gutter view through the anterolateral portal. From the anteromedial portal view with the knee in extension, the DLM is shifted posteriorly, and connective tissue is observed between the meniscus and the capsule-like ramp lesion. The DLM is reduced to the anterior position, and redundant connective tissue is observed as the knee is ranged from extension to flexion. The connective tissue is easily removed with an arthroscopic shaver, and the tear becomes more apparent. Saucerization technique starts from the border between the anterior horn and central area of the DLM with a 45° punch introduced from the anteromedial portal parallel to the circumferential fibers of the anterior horn of the DLM while viewing from the anterolateral portal. Measurement of the resection length is performed with a ruler from the anteromedial portal. After a 1-cm resection length is confirmed by viewing from the anterolateral portal, saucerization with removal of the central area is performed with a punch using the anteromedial portal. After the width of the remaining anterior, body and posterior horn of the lateral meniscus is confirmed to be 10 mm, sutures with 2-0 FiberWire are then passed through the anterior horn using a Scorpion suture passer through the anterolateral portal. Subsequently, a NanoPass is introduced through the anterolateral portal and penetrates the lower side of the capsule to reach the tibial side of the DLM and retrieve the lower side of the suture. Next, the same procedure is performed to penetrate the upper side of the capsule to reach the femoral side of the DLM and retrieve the upper side of the suture. After the suture is tied using a sliding knot technique and secured with a knot pusher, the entire process is then repeated. The ultimate number of sutures is determined based on the length of the tear and number needed for stabilization. The distance between individual sutures is 3 mm. After stabilization of the anterior meniscus, the posterior meniscus is often pulled anteriorly. After resection, the length is remeasured, and the posterior portion is resected until reaching a point 10 mm from the hiatus. Meniscal instability is again evaluated with a probe, and if instability persists in the posterior aspect, an arthroscopic rasp can be used to freshen the sites around the hiatus. The dual meniscal repair needles loaded with 2-0 braided polyester sutures are inserted into the unstable portion of the meniscus, including the horizontal tear through the cannula positioned in the anteromedial portal. The suture needles are retrieved under direct visualization through a previously prepared lateral incision. The sutures are tied over the capsule after every four sutures had been passed. Stitches are placed at 3-mm intervals. After repairing the posterior portion, the width of the repaired meniscus was confirmed to be 10 mm from the hiatus, and stability of the meniscus is confirmed by viewing from the anterolateral portal and with a McMurray test from the anteromedial portal view.

## References

[1] Yamasaki S., Hashimoto Y., Takigami J. (2017). Risk factors associated with knee joint degeneration after arthroscopic reshaping for juvenile discoid lateral meniscus. Am J Sports Med.

[2] Hashimoto Y, Nishino K, Yamasaki S, Nishida Y, Nakamura H. Two positioned MRI can visualize and detect the location of peripheral rim instability with snapping knee in the no-shift-type of complete discoid lateral meniscus [published online September 6, 2021]. *Arch Orthop Trauma Surg**.* https://doi.org/10.1007/s00402-021-04148-9 10.1007/s00402-021-04148-9 34487239

[3] Li Z., Zhao H., Dai Z. (2020). Widening of the popliteal hiatus on magnetic resonance imaging leads to recurrent subluxation of the lateral meniscus. Knee Surg Sports Traumatol Arthrosc.

[4] Hashimoto Y., Nishino K., Yamasaki S., Nishida Y., Takahashi S., Nakamura H. (2021). Predictive signs of peripheral rim instability with magnetic resonance imaging in no-shift-type complete discoid lateral meniscus. Skeletal Radiol.

[5] Restrepo R., Weisberg M.D., Pevsner R., Swirsky S., Lee E.Y. (2019). Discoid meniscus in the pediatric population: Emphasis on MR imaging signs of instability. Magn Reson Imaging Clin N Am.

[6] Hashimoto Y., Kazuya N., Takigami J. (2020). Abnormal displacement of discoid lateral meniscus with snapping knee detected by full extension and deep flexion MRI: Report of two cases. Asia Pac J Sports Med Arthrosc Rehabil Technol.

[7] Brophy R.H., Steinmetz R.G., Smith M.V., Matava M.J. (2022). Meniscal ramp lesions: Anatomy, epidemiology, diagnosis, and treatment. J Am Acad Orthop Surg.

[8] Hashimoto Y., Yamasaki S., Reid J.B., Guttmann D., Nishino K., Nakamura H. (2021). Arthroscopic saucerization with inside-out repair and anterocentral shift of a discoid lateral meniscus with retention of adequate volume of residual meniscus. Arthrosc Tech.

[9] Perkins C.A., Busch M.T., Christino M.A., Willimon S.C. (2021). Saucerization and repair of discoid lateral menisci with peripheral rim instability: Intermediate-term outcomes in children and adolescents. J Pediatr Orthop.

[10] Nishino K., Hashimoto Y., Tsumoto S., Yamasaki S., Nakamura H. (2021). Morphological changes in the residual meniscus after reshaping surgery for a discoid lateral meniscus. Am J Sports Med.

[11] Silberberg Muiño J.M., Nilo Fulvi A., Gimenez M., Muina Rullan J.R. (2018). Outside-in single-lasso loop technique for meniscal repair: Fast, economic, and reproducible. Arthrosc Tech.

[12] Muniandy M., Rajagopal S., Tahir S.H. (2019). Arthroscopic all-inside repair of tear of the anterior horn of discoid lateral meniscus. Surg J (N Y).

