# Supplementary material for: Surgical Management of Discoid Lateral Meniscus With Anterior Peripheral Instability: Retaining an Adequate Residual Meniscus Volume
Source: Arthrosc Tech. 2022 Jun 8;11(7):e1141–7. doi: 10.1016/j.eats.2022.02.021 (PMC9353039; doi:10.1016/j.eats.2022.02.021)
Supplement: Full ICMJE author disclosure forms [file mmc1.pdf]

Arthroscopy: The Journal of Arthroscopic and Related Surgery

— Instructions —

ICMJE Form for Disclosure of Potential Conflicts of Interest

Each author of the manuscript must separately complete and save this form using his or her name in the file name. Each author's completed form must then be uploaded with the manuscript.

The purpose of this form is to provide readers of your manuscript with information about your other interests that could influence how they receive and understand your work. The form is in four parts:

Section 1. Identifying information

Enter your full name and provide the manuscript title.

Section 2. The work under consideration for publication

This section asks for information about the work that you have submitted for publication. The time frame for this reporting is that of the work itself, from the initial conception and planning to the present. The requested information is about resources that you received, either directly or indirectly (via your institution), to enable you to complete the work. Checking "No" means that you did the work without receiving any financial support from any third party — that is, the work was supported by funds from the same institution that pays your salary and that institution did not receive third-party funds with which to pay you. If you or your institution received funds from a third party to support the work, such as a government granting agency, charitable foundation, or commercial sponsor, check "Yes." Then complete the provide the information requested.

Section 3. Relevant financial activities outside the submitted work

This section asks about your financial relationships with entities in the biomedical arena that could be perceived to influence, or that give the appearance of potentially influencing, what you wrote in the submitted work. You should disclose interactions with ANY entity that could be considered broadly relevant to the work.

Report all sources of revenue paid (or promised to be paid) directly to you or your institution on your behalf over the 36 months prior to submission of the work. This should include all monies from sources with relevance to the submitted work, not just monies from the entity that sponsored the research. Please note that your interactions with the work's sponsor that are outside the submitted work should also be listed here. If there is any question, it is usually better to disclose a relationship than not to do so.

For grants you have received for work outside the submitted work, you should disclose support ONLY from entities that could be perceived to be affected financially by the published work, such as entities or foundations supported by entities that could be perceived to have a financial stake in the outcome. Public funding sources, such as government agencies, charitable foundations, or academic institutions, need not be disclosed. For example, if a government agency sponsored a study in which you have been involved and drugs were provided by a pharmaceutical company, you need only list the pharmaceutical company.

Section 4. Other relationships

Use this section to report other relationships or activities that readers could perceive to have influenced, or that give the appearance of potentially influencing, what you wrote in the submitted work.

ICMJE Form for Disclosure of Potential Conflicts of Interest

Section 1. Identifying Information

1. Given Name ______Yusuke__________ 2. Surname Hashimoto__________________
3. Are you the corresponding author? Yes _x__ No___
4. Effective Date _____16/01/2022______________________
5. Manuscript Title ______Surgical management of discoid lateral meniscus with anterior peripheral instability: Retaining an adequate residual meniscus volume _______

Section 2. The Work Under Consideration for Publication

Did you or your institution at any time receive payment or services from a third party for any aspect of the submitted work (including but not limited to grants, data monitoring board, study design, manuscript preparation, statistical analysis, etc…)?

Complete each item by typing an X in answer yes or not and completing the information requested if an answer is Yes. If you have more than one relationship, add lines.

1. Grant

_ x_No ___Yes, money paid to you __Yes, money paid to institution* Name of entity___ Comments†___

2. Consulting fee or honorarium

_ x No ___Yes, money paid to you ___Yes, money paid to institution* Name of entity___ Comments†___

3. Support for travel to meetings for the study or other purposes

_ x No ___Yes, money paid to you ___Yes, money paid to institution* Name of entity___ Comments†___

4. Fees for participation in review activities such as data monitoring boards, statistical analysis, end-point committees, and the like

_ x No ___Yes, money paid to you ___Yes, money paid to institution* Name of entity___ Comments†___

5. Payment for writing or reviewing the manuscript

_ x No ___Yes, money paid to you ___Yes, money paid to institution* Name of entity___ Comments†___

6. Provision of writing assistance, medicines, equipment, or administrative support

_ x No ___Yes, money paid to you ___Yes, money paid to institution* Name of entity___ Comments†___

7. Other

_ x No ___Yes, money paid to you ___Yes, money paid to institution* Name of entity___ Comments†___

* This means money that your institution received for your efforts on this study.
† Use this section to provide any needed explanation.

Section 3. Relevant financial activities outside the submitted work

1. Board membership

_ x No ___Yes, money paid to you ___Yes, money paid to institution* Name of entity___ Comments___

2. Consultancy

_ x No ___Yes, money paid to you ___Yes, money paid to institution* Name of entity___ Comments___

3. Employment

_ x No ___Yes, money paid to you ___Yes, money paid to institution* Name of entity___ Comments___

4. Expert testimony

_ x No ___Yes, money paid to you ___Yes, money paid to institution* Name of entity___ Comments___

5. Grants/grants pending

_ x No ___Yes, money paid to you ___Yes, money paid to institution* Name of entity___ Comments___

6. Payment for lectures including service on speakers bureaus

_ x No ___Yes, money paid to you ___Yes, money paid to institution* Name of entity___ Comments___

7. Payment for manuscript preparation

_ x No ___Yes, money paid to you ___Yes, money paid to institution* Name of entity___ Comments___

8. Patents (planned, pending or issued)

_ x No ___Yes, money paid to you ___Yes, money paid to institution* Name of entity___ Comments___

9. Royalties

_ x No ___Yes, money paid to you ___Yes, money paid to institution* Name of entity___ Comments___

10. Payment for development of educational presentations

_ x No ___Yes, money paid to you ___Yes, money paid to institution* Name of entity___ Comments___

11. Stock/stock options

_ x No ___Yes, money paid to you ___Yes, money paid to institution* Name of entity___ Comments___

12. Travel/accommodations/ meeting expenses unrelated to activities listed**

_ x No ___Yes, money paid to you ___Yes, money paid to institution* Name of entity___ Comments___

13. Other (err on the side of full disclosure)

_ x No ___Yes, money paid to you ___Yes, money paid to institution* Name of entity___ Comments___

** For example, if you report a consultancy above there is no need to report travel related to that consultancy on this line.

Section 4. Other relationships

Are there other relationships or activities that readers could perceive to have influenced, or that give the appearance of potentially influencing, what you wrote in the submitted work?

_ x No other relationships/conditions/circumstances that present a potential conflict of interest

__Yes, the following relationships/conditions/circumstances are present (explain below):

>>>>>>>>>>><<<<<<<<<<<

The International Committee of Medical Journal Editors

The ICMJE Disclosure of Potential Conflicts of Interest Form was adopted by *Arthroscopy: The Journal of Arthroscopic and Related Surgery* along with 17 other leading orthopaedic journals at the 2011 annual meeting of the American Academy of Orthopaedic Surgeons.

Arthroscopy: The Journal of Arthroscopic and Related Surgery

— Instructions —

ICMJE Form for Disclosure of Potential Conflicts of Interest

Each author of the manuscript must separately complete and save this form using his or her name in the file name. Each author's completed form must then be uploaded with the manuscript.

The purpose of this form is to provide readers of your manuscript with information about your other interests that could influence how they receive and understand your work. The form is in four parts:

Section 1. Identifying information

Enter your full name and provide the manuscript title.

Section 2. The work under consideration for publication

This section asks for information about the work that you have submitted for publication. The time frame for this reporting is that of the work itself, from the initial conception and planning to the present. The requested information is about resources that you received, either directly or indirectly (via your institution), to enable you to complete the work. Checking "No" means that you did the work without receiving any financial support from any third party — that is, the work was supported by funds from the same institution that pays your salary and that institution did not receive third-party funds with which to pay you. If you or your institution received funds from a third party to support the work, such as a government granting agency, charitable foundation, or commercial sponsor, check "Yes." Then complete the provide the information requested.

Section 3. Relevant financial activities outside the submitted work

This section asks about your financial relationships with entities in the biomedical arena that could be perceived to influence, or that give the appearance of potentially influencing, what you wrote in the submitted work. You should disclose interactions with ANY entity that could be considered broadly relevant to the work.

Report all sources of revenue paid (or promised to be paid) directly to you or your institution on your behalf over the 36 months prior to submission of the work. This should include all monies from sources with relevance to the submitted work, not just monies from the entity that sponsored the research. Please note that your interactions with the work's sponsor that are outside the submitted work should also be listed here. If there is any question, it is usually better to disclose a relationship than not to do so.

For grants you have received for work outside the submitted work, you should disclose support ONLY from entities that could be perceived to be affected financially by the published work, such as entities or foundations supported by entities that could be perceived to have a financial stake in the outcome. Public funding sources, such as government agencies, charitable foundations, or academic institutions, need not be disclosed. For example, if a government agency sponsored a study in which you have been involved and drugs were provided by a pharmaceutical company, you need only list the pharmaceutical company.

Section 4. Other relationships

Use this section to report other relationships or activities that readers could perceive to have influenced, or that give the appearance of potentially influencing, what you wrote in the submitted work.

ICMJE Form for Disclosure of Potential Conflicts of Interest

Section 1. Identifying Information

1. Given Name ______Shinya______ 2. Surname ______Yamasaki______
3. Are you the corresponding author? Yes _ __ No_ x __
4. Effective Date _____21/01/2022____________________
5. Manuscript Title ______Surgical management of discoid lateral meniscus with anterior peripheral instability: Retaining an adequate residual meniscus volume _______

Section 2. The Work Under Consideration for Publication

Did you or your institution at any time receive payment or services from a third party for any aspect of the submitted work (including but not limited to grants, data monitoring board, study design, manuscript preparation, statistical analysis, etc…)?

Complete each item by typing an X in answer yes or not and completing the information requested if an answer is Yes. If you have more than one relationship, add lines.

1. Grant

_ x_No ___Yes, money paid to you __Yes, money paid to institution* Name of entity___ Comments†___

2. Consulting fee or honorarium

_ x No ___Yes, money paid to you ___Yes, money paid to institution* Name of entity___ Comments†___

3. Support for travel to meetings for the study or other purposes

_ x No ___Yes, money paid to you ___Yes, money paid to institution* Name of entity___ Comments†___

4. Fees for participation in review activities such as data monitoring boards, statistical analysis, end-point committees, and the like

_ x No ___Yes, money paid to you ___Yes, money paid to institution* Name of entity___ Comments†___

5. Payment for writing or reviewing the manuscript

_ x No ___Yes, money paid to you ___Yes, money paid to institution* Name of entity___ Comments†___

6. Provision of writing assistance, medicines, equipment, or administrative support

_ x No ___Yes, money paid to you ___Yes, money paid to institution* Name of entity___ Comments†___

7. Other

_ x No ___Yes, money paid to you ___Yes, money paid to institution* Name of entity___ Comments†___

* This means money that your institution received for your efforts on this study.
† Use this section to provide any needed explanation.

Section 3. Relevant financial activities outside the submitted work

1. Board membership

_ x No ___Yes, money paid to you ___Yes, money paid to institution* Name of entity___ Comments___

2. Consultancy

_ x No ___Yes, money paid to you ___Yes, money paid to institution* Name of entity___ Comments___

3. Employment

_ x No ___Yes, money paid to you ___Yes, money paid to institution* Name of entity___ Comments___

4. Expert testimony

_ x No ___Yes, money paid to you ___Yes, money paid to institution* Name of entity___ Comments___

5. Grants/grants pending

_ x No ___Yes, money paid to you ___Yes, money paid to institution* Name of entity___ Comments___

6. Payment for lectures including service on speakers bureaus

_ x No ___Yes, money paid to you ___Yes, money paid to institution* Name of entity___ Comments___

7. Payment for manuscript preparation

_ x No ___Yes, money paid to you ___Yes, money paid to institution* Name of entity___ Comments___

8. Patents (planned, pending or issued)

_ x No ___Yes, money paid to you ___Yes, money paid to institution* Name of entity___ Comments___

9. Royalties

_ x No ___Yes, money paid to you ___Yes, money paid to institution* Name of entity___ Comments___

10. Payment for development of educational presentations

_ x No ___Yes, money paid to you ___Yes, money paid to institution* Name of entity___ Comments___

11. Stock/stock options

_ x No ___Yes, money paid to you ___Yes, money paid to institution* Name of entity___ Comments___

12. Travel/accommodations/ meeting expenses unrelated to activities listed**

_ x No ___Yes, money paid to you ___Yes, money paid to institution* Name of entity___ Comments___

13. Other (err on the side of full disclosure)

_ x No ___Yes, money paid to you ___Yes, money paid to institution* Name of entity___ Comments___

** For example, if you report a consultancy above there is no need to report travel related to that consultancy on this line.

Section 4. Other relationships

Are there other relationships or activities that readers could perceive to have influenced, or that give the appearance of potentially influencing, what you wrote in the submitted work?

_ x No other relationships/conditions/circumstances that present a potential conflict of interest

__Yes, the following relationships/conditions/circumstances are present (explain below):

>>>>>>>>>>><<<<<<<<<<<

The International Committee of Medical Journal Editors

The ICMJE Disclosure of Potential Conflicts of Interest Form was adopted by *Arthroscopy: The Journal of Arthroscopic and Related Surgery* along with 17 other leading orthopaedic journals at the 2011 annual meeting of the American Academy of Orthopaedic Surgeons.

Arthroscopy: The Journal of Arthroscopic and Related Surgery

— Instructions —

ICMJE Form for Disclosure of Potential Conflicts of Interest

Each author of the manuscript must separately complete and save this form using his or her name in the file name. Each author's completed form must then be uploaded with the manuscript.

The purpose of this form is to provide readers of your manuscript with information about your other interests that could influence how they receive and understand your work. The form is in four parts:

Section 1. Identifying information

Enter your full name and provide the manuscript title.

Section 2. The work under consideration for publication

This section asks for information about the work that you have submitted for publication. The time frame for this reporting is that of the work itself, from the initial conception and planning to the present. The requested information is about resources that you received, either directly or indirectly (via your institution), to enable you to complete the work. Checking "No" means that you did the work without receiving any financial support from any third party — that is, the work was supported by funds from the same institution that pays your salary and that institution did not receive third-party funds with which to pay you. If you or your institution received funds from a third party to support the work, such as a government granting agency, charitable foundation, or commercial sponsor, check "Yes." Then complete the provide the information requested.

Section 3. Relevant financial activities outside the submitted work

This section asks about your financial relationships with entities in the biomedical arena that could be perceived to influence, or that give the appearance of potentially influencing, what you wrote in the submitted work. You should disclose interactions with ANY entity that could be considered broadly relevant to the work.

Report all sources of revenue paid (or promised to be paid) directly to you or your institution on your behalf over the 36 months prior to submission of the work. This should include all monies from sources with relevance to the submitted work, not just monies from the entity that sponsored the research. Please note that your interactions with the work's sponsor that are outside the submitted work should also be listed here. If there is any question, it is usually better to disclose a relationship than not to do so.

For grants you have received for work outside the submitted work, you should disclose support ONLY from entities that could be perceived to be affected financially by the published work, such as entities or foundations supported by entities that could be perceived to have a financial stake in the outcome. Public funding sources, such as government agencies, charitable foundations, or academic institutions, need not be disclosed. For example, if a government agency sponsored a study in which you have been involved and drugs were provided by a pharmaceutical company, you need only list the pharmaceutical company.

Section 4. Other relationships

Use this section to report other relationships or activities that readers could perceive to have influenced, or that give the appearance of potentially influencing, what you wrote in the submitted work.

ICMJE Form for Disclosure of Potential Conflicts of Interest

Section 1. Identifying Information

1. Given Name ____Dan__________________ 2. Surname ___Guttmann___________________________
3. Are you the corresponding author? Yes ___ No_X__
4. Effective Date _12_/ 01 /2021___________________________
5. Manuscript Title _____Surgical management of discoid lateral meniscus with anterior peripheral instability: Retaining an adequate residual meniscus volume ______

Section 2. The Work Under Consideration for Publication

Did you or your institution at any time receive payment or services from a third party for any aspect of the submitted work (including but not limited to grants, data monitoring board, study design, manuscript preparation, statistical analysis, etc...)?

Complete each item by typing an X in answer yes or not and completing the information requested if an answer is Yes. If you have more than one relationship, add lines.

1. Grant

X__No ___Yes, money paid to you ___Yes, money paid to institution* Name of entity___ Comments†___

2. Consulting fee or honorarium

X__No ___Yes, money paid to you ___Yes, money paid to institution* Name of entity___ Comments†___

3. Support for travel to meetings for the study or other purposes

X__No ___Yes, money paid to you ___Yes, money paid to institution* Name of entity___ Comments†___

4. Fees for participation in review activities such as data monitoring boards, statistical analysis, end-point committees, and the like

X__No ___Yes, money paid to you ___Yes, money paid to institution* Name of entity___ Comments†___

5. Payment for writing or reviewing the manuscript

X__No ___Yes, money paid to you ___Yes, money paid to institution* Name of entity___ Comments†___

6. Provision of writing assistance, medicines, equipment, or administrative support

X__No ___Yes, money paid to you ___Yes, money paid to institution* Name of entity___ Comments†___

7. Other

X__No ___Yes, money paid to you ___Yes, money paid to institution* Name of entity___ Comments†___

* This means money that your institution received for your efforts on this study.
† Use this section to provide any needed explanation.

Section 3. Relevant financial activities outside the submitted work

1. Board membership

X_No ___Yes, money paid to you ___Yes, money paid to institution* Name of entity___ Comments___

2. Consultancy

__No ___Yes, money paid to you ___Yes, money paid to institution* Name of entity___ Comments Arthrex Consultant No money paid for this paper and related research__X_

3. Employment

X__No ___Yes, money paid to you ___Yes, money paid to institution* Name of entity___ Comments___

4. Expert testimony

X__No ___Yes, money paid to you ___Yes, money paid to institution* Name of entity___ Comments___

5. Grants/grants pending

X__No ___Yes, money paid to you ___Yes, money paid to institution* Name of entity___ Comments___

6. Payment for lectures including service on speakers bureaus

__No X ___Yes, money paid to you ___Yes, money paid to institution* Name of entity___ Comments__Unrelated to this paper and associated research_

7. Payment for manuscript preparation

X__No ___Yes, money paid to you ___Yes, money paid to institution* Name of entity___ Comments___

8. Patents (planned, pending or issued)

X__No ___Yes, money paid to you ___Yes, money paid to institution* Name of entity___ Comments___

9. Royalties

X__No ___Yes, money paid to you ___Yes, money paid to institution* Name of entity___ Comments___

10. Payment for development of educational presentations

__No X___Yes, money paid to you ___Yes, money paid to institution* Name of entity___ Comments__Arthrex Consultant unrelated to this paper and associated research_

11. Stock/stock options

X__No ___Yes, money paid to you ___Yes, money paid to institution* Name of entity___ Comments___

12. Travel/accommodations/ meeting expenses unrelated to activities listed**

X__No ___Yes, money paid to you ___Yes, money paid to institution* Name of entity___ Comments___

13. Other (err on the side of full disclosure)

X__No ___Yes, money paid to you ___Yes, money paid to institution* Name of entity___ Comments___

I am an Arthrex Consultant unrelated to this paper/manuscript and associated research

** For example, if you report a consultancy above there is no need to report travel related to that consultancy on this line.

Section 4. Other relationships

Are there other relationships or activities that readers could perceive to have influenced, or that give the appearance of potentially influencing, what you wrote in the submitted work?

_x_No other relationships/conditions/circumstances that present a potential conflict of interest

__Yes, the following relationships/conditions/circumstances are present (explain below):

>>>>>>>>>>><<<<<<<<<<<

The International Committee of Medical Journal Editors

The ICMJE Disclosure of Potential Conflicts of Interest Form was adopted by *Arthroscopy: The Journal of Arthroscopic and Related Surgery* along with 17 other leading orthopaedic journals at the 2011 annual meeting of the American Academy of Orthopaedic Surgeons.

Arthroscopy: The Journal of Arthroscopic and Related Surgery

— Instructions —

ICMJE Form for Disclosure of Potential Conflicts of Interest

Each author of the manuscript must separately complete and save this form using his or her name in the file name. Each author's completed form must then be uploaded with the manuscript.

The purpose of this form is to provide readers of your manuscript with information about your other interests that could influence how they receive and understand your work. The form is in four parts:

Section 1. Identifying information

Enter your full name and provide the manuscript title.

Section 2. The work under consideration for publication

This section asks for information about the work that you have submitted for publication. The time frame for this reporting is that of the work itself, from the initial conception and planning to the present. The requested information is about resources that you received, either directly or indirectly (via your institution), to enable you to complete the work. Checking "No" means that you did the work without receiving any financial support from any third party — that is, the work was supported by funds from the same institution that pays your salary and that institution did not receive third-party funds with which to pay you. If you or your institution received funds from a third party to support the work, such as a government granting agency, charitable foundation, or commercial sponsor, check "Yes." Then complete the provide the information requested.

Section 3. Relevant financial activities outside the submitted work

This section asks about your financial relationships with entities in the biomedical arena that could be perceived to influence, or that give the appearance of potentially influencing, what you wrote in the submitted work. You should disclose interactions with ANY entity that could be considered broadly relevant to the work.

Report all sources of revenue paid (or promised to be paid) directly to you or your institution on your behalf over the 36 months prior to submission of the work. This should include all monies from sources with relevance to the submitted work, not just monies from the entity that sponsored the research. Please note that your interactions with the work's sponsor that are outside the submitted work should also be listed here. If there is any question, it is usually better to disclose a relationship than not to do so.

For grants you have received for work outside the submitted work, you should disclose support ONLY from entities that could be perceived to be affected financially by the published work, such as entities or foundations supported by entities that could be perceived to have a financial stake in the outcome. Public funding sources, such as government agencies, charitable foundations, or academic institutions, need not be disclosed. For example, if a government agency sponsored a study in which you have been involved and drugs were provided by a pharmaceutical company, you need only list the pharmaceutical company.

Section 4. Other relationships

Use this section to report other relationships or activities that readers could perceive to have influenced, or that give the appearance of potentially influencing, what you wrote in the submitted work.

ICMJE Form for Disclosure of Potential Conflicts of Interest

Section 1. Identifying Information

1. Given Name ____John__________________ 2. Surname ____Reid__III________________________
3. Are you the corresponding author? Yes ___ No_X__
4. Effective Date __1/1/2021__________________________
5. Manuscript Title ______Surgical management of discoid lateral meniscus with anterior peripheral instability: Retaining an adequate residual meniscus volume ______

Section 2. The Work Under Consideration for Publication

Did you or your institution at any time receive payment or services from a third party for any aspect of the submitted work (including but not limited to grants, data monitoring board, study design, manuscript preparation, statistical analysis, etc…)?

Complete each item by typing an X in answer yes or not and completing the information requested if an answer is Yes. If you have more than one relationship, add lines.

1. Grant

X__No ___Yes, money paid to you ___Yes, money paid to institution* Name of entity___ Comments†___

2. Consulting fee or honorarium

X__No ___Yes, money paid to you ___Yes, money paid to institution* Name of entity___ Comments†___

3. Support for travel to meetings for the study or other purposes

X__No ___Yes, money paid to you ___Yes, money paid to institution* Name of entity___ Comments†___

4. Fees for participation in review activities such as data monitoring boards, statistical analysis, end-point committees, and the like

X__No ___Yes, money paid to you ___Yes, money paid to institution* Name of entity___ Comments†___

5. Payment for writing or reviewing the manuscript

X__No ___Yes, money paid to you ___Yes, money paid to institution* Name of entity___ Comments†___

6. Provision of writing assistance, medicines, equipment, or administrative support

X__No ___Yes, money paid to you ___Yes, money paid to institution* Name of entity___ Comments†___

7. Other

X__No ___Yes, money paid to you ___Yes, money paid to institution* Name of entity___ Comments†___

* This means money that your institution received for your efforts on this study.
† Use this section to provide any needed explanation.

Section 3. Relevant financial activities outside the submitted work

1. Board membership

X_No ___Yes, money paid to you ___Yes, money paid to institution* Name of entity___ Comments___

2. Consultancy

__No X___Yes, money paid to you ___Yes, money paid to institution* Name of entity___ Comments Arthrex Consultant No money paid for this paper and related research___

3. Employment

__No _X__Yes, money paid to you ___Yes, money paid to institution* Name of entity Asante___ Comments_Employed orthopaedic surgeon No money paid for this paper and related research__

4. Expert testimony

X__No ___Yes, money paid to you ___Yes, money paid to institution* Name of entity___ Comments___

5. Grants/grants pending

X__No ___Yes, money paid to you ___Yes, money paid to institution* Name of entity___ Comments___

6. Payment for lectures including service on speakers bureaus

__No X ___Yes, money paid to you ___Yes, money paid to institution* Name of entity___ Comments__Unrelated to this paper and associated research_

7. Payment for manuscript preparation

X__No ___Yes, money paid to you ___Yes, money paid to institution* Name of entity___ Comments___

8. Patents (planned, pending or issued)

X__No ___Yes, money paid to you ___Yes, money paid to institution* Name of entity___ Comments___

9. Royalties

X__No ___Yes, money paid to you ___Yes, money paid to institution* Name of entity___ Comments___

10. Payment for development of educational presentations

__No X___Yes, money paid to you ___Yes, money paid to institution* Name of entity___ Comments__Arthrex Consultant unrelated to this paper and associated research_

11. Stock/stock options

X__No ___Yes, money paid to you ___Yes, money paid to institution* Name of entity___ Comments___

12. Travel/accommodations/ meeting expenses unrelated to activities listed**

X__No ___Yes, money paid to you ___Yes, money paid to institution* Name of entity___ Comments___

13. Other (err on the side of full disclosure)

X__No ___Yes, money paid to you ___Yes, money paid to institution* Name of entity___ Comments___

I am an Arthrex Consultant unrelated to this paper/manuscript and associated research

** For example, if you report a consultancy above there is no need to report travel related to that consultancy on this line.

Section 4. Other relationships

Are there other relationships or activities that readers could perceive to have influenced, or that give the appearance of potentially influencing, what you wrote in the submitted work?

X__No other relationships/conditions/circumstances that present a potential conflict of interest

__Yes, the following relationships/conditions/circumstances are present (explain below):

>>>>>>>>>>><<<<<<<<<<<

The International Committee of Medical Journal Editors

The ICMJE Disclosure of Potential Conflicts of Interest Form was adopted by *Arthroscopy: The Journal of Arthroscopic and Related Surgery* along with 17 other leading orthopaedic journals at the 2011 annual meeting of the American Academy of Orthopaedic Surgeons.

Arthroscopy: The Journal of Arthroscopic and Related Surgery

— Instructions —

ICMJE Form for Disclosure of Potential Conflicts of Interest

Each author of the manuscript must separately complete and save this form using his or her name in the file name. Each author's completed form must then be uploaded with the manuscript.

The purpose of this form is to provide readers of your manuscript with information about your other interests that could influence how they receive and understand your work. The form is in four parts:

Section 1. Identifying information

Enter your full name and provide the manuscript title.

Section 2. The work under consideration for publication

This section asks for information about the work that you have submitted for publication. The time frame for this reporting is that of the work itself, from the initial conception and planning to the present. The requested information is about resources that you received, either directly or indirectly (via your institution), to enable you to complete the work. Checking "No" means that you did the work without receiving any financial support from any third party — that is, the work was supported by funds from the same institution that pays your salary and that institution did not receive third-party funds with which to pay you. If you or your institution received funds from a third party to support the work, such as a government granting agency, charitable foundation, or commercial sponsor, check "Yes." Then complete the provide the information requested.

Section 3. Relevant financial activities outside the submitted work

This section asks about your financial relationships with entities in the biomedical arena that could be perceived to influence, or that give the appearance of potentially influencing, what you wrote in the submitted work. You should disclose interactions with ANY entity that could be considered broadly relevant to the work.

Report all sources of revenue paid (or promised to be paid) directly to you or your institution on your behalf over the 36 months prior to submission of the work. This should include all monies from sources with relevance to the submitted work, not just monies from the entity that sponsored the research. Please note that your interactions with the work's sponsor that are outside the submitted work should also be listed here. If there is any question, it is usually better to disclose a relationship than not to do so.

For grants you have received for work outside the submitted work, you should disclose support ONLY from entities that could be perceived to be affected financially by the published work, such as entities or foundations supported by entities that could be perceived to have a financial stake in the outcome. Public funding sources, such as government agencies, charitable foundations, or academic institutions, need not be disclosed. For example, if a government agency sponsored a study in which you have been involved and drugs were provided by a pharmaceutical company, you need only list the pharmaceutical company.

Section 4. Other relationships

Use this section to report other relationships or activities that readers could perceive to have influenced, or that give the appearance of potentially influencing, what you wrote in the submitted work.

ICMJE Form for Disclosure of Potential Conflicts of Interest

Section 1. Identifying Information

1. Given Name ____Sean__________________ 2. Surname ____Marvil__________________________
3. Are you the corresponding author? Yes ___ No_X__
4. Effective Date 12 /30/2021___________________________
5. Manuscript Title _____Surgical management of discoid lateral meniscus with anterior peripheral instability: Retaining an adequate residual meniscus volume______

Section 2. The Work Under Consideration for Publication

Did you or your institution at any time receive payment or services from a third party for any aspect of the submitted work (including but not limited to grants, data monitoring board, study design, manuscript preparation, statistical analysis, etc…)?

Complete each item by typing an X in answer yes or not and completing the information requested if an answer is Yes. If you have more than one relationship, add lines.

1. Grant

X__No ___Yes, money paid to you ___Yes, money paid to institution* Name of entity___ Comments†___

2. Consulting fee or honorarium

X__No ___Yes, money paid to you ___Yes, money paid to institution* Name of entity___ Comments†___

3. Support for travel to meetings for the study or other purposes

X__No ___Yes, money paid to you ___Yes, money paid to institution* Name of entity___ Comments†___

4. Fees for participation in review activities such as data monitoring boards, statistical analysis, end-point committees, and the like

X__No ___Yes, money paid to you ___Yes, money paid to institution* Name of entity___ Comments†___

5. Payment for writing or reviewing the manuscript

X__No ___Yes, money paid to you ___Yes, money paid to institution* Name of entity___ Comments†___

6. Provision of writing assistance, medicines, equipment, or administrative support

X__No ___Yes, money paid to you ___Yes, money paid to institution* Name of entity___ Comments†___

7. Other

X__No ___Yes, money paid to you ___Yes, money paid to institution* Name of entity___ Comments†___

* This means money that your institution received for your efforts on this study.
† Use this section to provide any needed explanation.

Section 3. Relevant financial activities outside the submitted work

1. Board membership

X__No ___Yes, money paid to you ___Yes, money paid to institution* Name of entity___ Comments___

2. Consultancy

__No X___Yes, money paid to you ___Yes, money paid to institution* Name of entity___ Comments Arthrex Consultant No money paid for this paper and related research___

3. Employment

X__No ___Yes, money paid to you ___Yes, money paid to institution* Name of entity___ Comments___

4. Expert testimony

X__No ___Yes, money paid to you ___Yes, money paid to institution* Name of entity___ Comments___

5. Grants/grants pending

X__No ___Yes, money paid to you ___Yes, money paid to institution* Name of entity___ Comments___

6. Payment for lectures including service on speakers bureaus

__No X ___Yes, money paid to you ___Yes, money paid to institution* Name of entity___ Comments__Unrelated to this paper and associated research_

7. Payment for manuscript preparation

X__No ___Yes, money paid to you ___Yes, money paid to institution* Name of entity___ Comments___

8. Patents (planned, pending or issued)

X__No ___Yes, money paid to you ___Yes, money paid to institution* Name of entity___ Comments___

9. Royalties

X__No ___Yes, money paid to you ___Yes, money paid to institution* Name of entity___ Comments___

10. Payment for development of educational presentations

__No X___Yes, money paid to you ___Yes, money paid to institution* Name of entity___ Comments__Arthrex Consultant unrelated to this paper and associated research_

11. Stock/stock options

X__No ___Yes, money paid to you ___Yes, money paid to institution* Name of entity___ Comments___

12. Travel/accommodations/ meeting expenses unrelated to activities listed**

X__No ___Yes, money paid to you ___Yes, money paid to institution* Name of entity___ Comments___

13. Other (err on the side of full disclosure)

X__No ___Yes, money paid to you ___Yes, money paid to institution* Name of entity___ Comments___

I am an Arthrex Consultant unrelated to this paper/manuscript and associated research

** For example, if you report a consultancy above there is no need to report travel related to that consultancy on this line.

Section 4. Other relationships

Are there other relationships or activities that readers could perceive to have influenced, or that give the appearance of potentially influencing, what you wrote in the submitted work?

X__No other relationships/conditions/circumstances that present a potential conflict of interest

__Yes, the following relationships/conditions/circumstances are present (explain below):

>>>>>>>>>>><<<<<<<<<<<

The International Committee of Medical Journal Editors

The ICMJE Disclosure of Potential Conflicts of Interest Form was adopted by *Arthroscopy: The Journal of Arthroscopic and Related Surgery* along with 17 other leading orthopaedic journals at the 2011 annual meeting of the American Academy of Orthopaedic Surgeons.

Arthroscopy: The Journal of Arthroscopic and Related Surgery

— Instructions —

ICMJE Form for Disclosure of Potential Conflicts of Interest

Each author of the manuscript must separately complete and save this form using his or her name in the file name. Each author's completed form must then be uploaded with the manuscript.

The purpose of this form is to provide readers of your manuscript with information about your other interests that could influence how they receive and understand your work. The form is in four parts:

Section 1. Identifying information

Enter your full name and provide the manuscript title.

Section 2. The work under consideration for publication

This section asks for information about the work that you have submitted for publication. The time frame for this reporting is that of the work itself, from the initial conception and planning to the present. The requested information is about resources that you received, either directly or indirectly (via your institution), to enable you to complete the work. Checking "No" means that you did the work without receiving any financial support from any third party — that is, the work was supported by funds from the same institution that pays your salary and that institution did not receive third-party funds with which to pay you. If you or your institution received funds from a third party to support the work, such as a government granting agency, charitable foundation, or commercial sponsor, check "Yes." Then complete the provide the information requested.

Section 3. Relevant financial activities outside the submitted work

This section asks about your financial relationships with entities in the biomedical arena that could be perceived to influence, or that give the appearance of potentially influencing, what you wrote in the submitted work. You should disclose interactions with ANY entity that could be considered broadly relevant to the work.

Report all sources of revenue paid (or promised to be paid) directly to you or your institution on your behalf over the 36 months prior to submission of the work. This should include all monies from sources with relevance to the submitted work, not just monies from the entity that sponsored the research. Please note that your interactions with the work's sponsor that are outside the submitted work should also be listed here. If there is any question, it is usually better to disclose a relationship than not to do so.

For grants you have received for work outside the submitted work, you should disclose support ONLY from entities that could be perceived to be affected financially by the published work, such as entities or foundations supported by entities that could be perceived to have a financial stake in the outcome. Public funding sources, such as government agencies, charitable foundations, or academic institutions, need not be disclosed. For example, if a government agency sponsored a study in which you have been involved and drugs were provided by a pharmaceutical company, you need only list the pharmaceutical company.

Section 4. Other relationships

Use this section to report other relationships or activities that readers could perceive to have influenced, or that give the appearance of potentially influencing, what you wrote in the submitted work.

ICMJE Form for Disclosure of Potential Conflicts of Interest

Section 1. Identifying Information

1. Given Name ___Takuya___ 2. Surname ___Kinoshita___
3. Are you the corresponding author? Yes _ __ No_ x __
4. Effective Date _____21/01/2022_____________________
5. Manuscript Title _____Surgical management of discoid lateral meniscus with anterior peripheral instability: Retaining an adequate residual meniscus volume_______

Section 2. The Work Under Consideration for Publication

Did you or your institution at any time receive payment or services from a third party for any aspect of the submitted work (including but not limited to grants, data monitoring board, study design, manuscript preparation, statistical analysis, etc…)?

Complete each item by typing an X in answer yes or not and completing the information requested if an answer is Yes. If you have more than one relationship, add lines.

1. Grant

_ x_No ___Yes, money paid to you __Yes, money paid to institution* Name of entity___ Comments†___

2. Consulting fee or honorarium

_ x No ___Yes, money paid to you ___Yes, money paid to institution* Name of entity___ Comments†___

3. Support for travel to meetings for the study or other purposes

_ x No ___Yes, money paid to you ___Yes, money paid to institution* Name of entity___ Comments†___

4. Fees for participation in review activities such as data monitoring boards, statistical analysis, end-point committees, and the like

_ x No ___Yes, money paid to you ___Yes, money paid to institution* Name of entity___ Comments†___

5. Payment for writing or reviewing the manuscript

_ x No ___Yes, money paid to you ___Yes, money paid to institution* Name of entity___ Comments†___

6. Provision of writing assistance, medicines, equipment, or administrative support

_ x No ___Yes, money paid to you ___Yes, money paid to institution* Name of entity___ Comments†___

7. Other

_ x No ___Yes, money paid to you ___Yes, money paid to institution* Name of entity___ Comments†___

* This means money that your institution received for your efforts on this study.
† Use this section to provide any needed explanation.

Section 3. Relevant financial activities outside the submitted work

1. Board membership

_ x No ___Yes, money paid to you ___Yes, money paid to institution* Name of entity___ Comments___

2. Consultancy

_ x No ___Yes, money paid to you ___Yes, money paid to institution* Name of entity___ Comments___

3. Employment

_ x No ___Yes, money paid to you ___Yes, money paid to institution* Name of entity___ Comments___

4. Expert testimony

_ x No ___Yes, money paid to you ___Yes, money paid to institution* Name of entity___ Comments___

5. Grants/grants pending

_ x No ___Yes, money paid to you ___Yes, money paid to institution* Name of entity___ Comments___

6. Payment for lectures including service on speakers bureaus

_ x No ___Yes, money paid to you ___Yes, money paid to institution* Name of entity___ Comments___

7. Payment for manuscript preparation

_ x No ___Yes, money paid to you ___Yes, money paid to institution* Name of entity___ Comments___

8. Patents (planned, pending or issued)

_ x No ___Yes, money paid to you ___Yes, money paid to institution* Name of entity___ Comments___

9. Royalties

_ x No ___Yes, money paid to you ___Yes, money paid to institution* Name of entity___ Comments___

10. Payment for development of educational presentations

_ x No ___Yes, money paid to you ___Yes, money paid to institution* Name of entity___ Comments___

11. Stock/stock options

_ x No ___Yes, money paid to you ___Yes, money paid to institution* Name of entity___ Comments___

12. Travel/accommodations/ meeting expenses unrelated to activities listed**

_ x No ___Yes, money paid to you ___Yes, money paid to institution* Name of entity___ Comments___

13. Other (err on the side of full disclosure)

_ x No ___Yes, money paid to you ___Yes, money paid to institution* Name of entity___ Comments___

** For example, if you report a consultancy above there is no need to report travel related to that consultancy on this line.

Section 4. Other relationships

Are there other relationships or activities that readers could perceive to have influenced, or that give the appearance of potentially influencing, what you wrote in the submitted work?

_ x No other relationships/conditions/circumstances that present a potential conflict of interest

__Yes, the following relationships/conditions/circumstances are present (explain below):

>>>>>>>>>>><<<<<<<<<<<

The International Committee of Medical Journal Editors

The ICMJE Disclosure of Potential Conflicts of Interest Form was adopted by *Arthroscopy: The Journal of Arthroscopic and Related Surgery* along with 17 other leading orthopaedic journals at the 2011 annual meeting of the American Academy of Orthopaedic Surgeons.

Arthroscopy: The Journal of Arthroscopic and Related Surgery

— Instructions —

ICMJE Form for Disclosure of Potential Conflicts of Interest

Each author of the manuscript must separately complete and save this form using his or her name in the file name. Each author's completed form must then be uploaded with the manuscript.

The purpose of this form is to provide readers of your manuscript with information about your other interests that could influence how they receive and understand your work. The form is in four parts:

Section 1. Identifying information

Enter your full name and provide the manuscript title.

Section 2. The work under consideration for publication

This section asks for information about the work that you have submitted for publication. The time frame for this reporting is that of the work itself, from the initial conception and planning to the present. The requested information is about resources that you received, either directly or indirectly (via your institution), to enable you to complete the work. Checking "No" means that you did the work without receiving any financial support from any third party — that is, the work was supported by funds from the same institution that pays your salary and that institution did not receive third-party funds with which to pay you. If you or your institution received funds from a third party to support the work, such as a government granting agency, charitable foundation, or commercial sponsor, check "Yes." Then complete the provide the information requested.

Section 3. Relevant financial activities outside the submitted work

This section asks about your financial relationships with entities in the biomedical arena that could be perceived to influence, or that give the appearance of potentially influencing, what you wrote in the submitted work. You should disclose interactions with ANY entity that could be considered broadly relevant to the work.

Report all sources of revenue paid (or promised to be paid) directly to you or your institution on your behalf over the 36 months prior to submission of the work. This should include all monies from sources with relevance to the submitted work, not just monies from the entity that sponsored the research. Please note that your interactions with the work's sponsor that are outside the submitted work should also be listed here. If there is any question, it is usually better to disclose a relationship than not to do so.

For grants you have received for work outside the submitted work, you should disclose support ONLY from entities that could be perceived to be affected financially by the published work, such as entities or foundations supported by entities that could be perceived to have a financial stake in the outcome. Public funding sources, such as government agencies, charitable foundations, or academic institutions, need not be disclosed. For example, if a government agency sponsored a study in which you have been involved and drugs were provided by a pharmaceutical company, you need only list the pharmaceutical company.

Section 4. Other relationships

Use this section to report other relationships or activities that readers could perceive to have influenced, or that give the appearance of potentially influencing, what you wrote in the submitted work.

ICMJE Form for Disclosure of Potential Conflicts of Interest

Section 1. Identifying Information

1. Given Name __Hiroaki__ 2. Surname __Nakamura__
3. Are you the corresponding author? Yes ___ No_X__
4. Effective Date ______20/01/2022____________________
5. Manuscript Title ____Surgical management of discoid lateral meniscus with anterior peripheral instability: Retaining an adequate residual meniscus volume _________

Section 2. The Work Under Consideration for Publication

Did you or your institution at any time receive payment or services from a third party for any aspect of the submitted work (including but not limited to grants, data monitoring board, study design, manuscript preparation, statistical analysis, etc…)?

Complete each item by typing an X in answer yes or not and completing the information requested if an answer is Yes. If you have more than one relationship, add lines.

1. Grant

_X_No ___Yes, money paid to you ___Yes, money paid to institution* Name of entity___ Comments†___

2. Consulting fee or honorarium

_X_No ___Yes, money paid to you ___Yes, money paid to institution* Name of entity___ Comments†___

3. Support for travel to meetings for the study or other purposes

_X_No ___Yes, money paid to you ___Yes, money paid to institution* Name of entity___ Comments†___

4. Fees for participation in review activities such as data monitoring boards, statistical analysis, end-point committees, and the like

_X_No ___Yes, money paid to you ___Yes, money paid to institution* Name of entity___ Comments†___

5. Payment for writing or reviewing the manuscript

_X_No ___Yes, money paid to you ___Yes, money paid to institution* Name of entity___ Comments†___

6. Provision of writing assistance, medicines, equipment, or administrative support

_X_No ___Yes, money paid to you ___Yes, money paid to institution* Name of entity___ Comments†___

7. Other

_X_No ___Yes, money paid to you ___Yes, money paid to institution* Name of entity___ Comments†___

* This means money that your institution received for your efforts on this study.
† Use this section to provide any needed explanation.

Section 3. Relevant financial activities outside the submitted work

1. Board membership

_X_No ___Yes, money paid to you ___Yes, money paid to institution* Name of entity___ Comments___

2. Consultancy

_X_No ___Yes, money paid to you ___Yes, money paid to institution* Name of entity___ Comments___

3. Employment

__No _X_Yes, money paid to you _X_Yes, money paid to institution* Name of entity_Osaka City University_ Comments___

3. Employment

__No _X_Yes, money paid to you _X_Yes, money paid to institution* Name of entity_Shiraniwa Hospital_ Comments___

4. Expert testimony

_X_No ___Yes, money paid to you ___Yes, money paid to institution* Name of entity___ Comments___

5. Grants/grants pending

__No _X_Yes, money paid to you _X_Yes, money paid to institution* Name of entity_Japan Society for the Promotion of Science_ Comments___

5. Grants/grants pending

__No _X_Yes, money paid to you _X_Yes, money paid to institution* Name of entity_Japan Agency for Medical Research and Development_ Comments___

6. Payment for lectures including service on speakers bureaus

__No _X_Yes, money paid to you _X_Yes, money paid to institution* Name of entity_DAIICHI SANKYO COMPANEY, LIMITED_ Comments___

6. Payment for lectures including service on speakers bureaus

__No _X_Yes, money paid to you _X_Yes, money paid to institution* Name of entity_Taisho Toyama Pharmaceutical Co., Ltd._ Comments___

6. Payment for lectures including service on speakers bureaus

__No _X_Yes, money paid to you _X_Yes, money paid to institution* Name of entity_SHIONOGI & CO., LTD._ Comments___

6. Payment for lectures including service on speakers bureaus

__No _X_Yes, money paid to you _X_Yes, money paid to institution* Name of entity_Eli Lilly Japan K.K_ Comments___

7. Payment for manuscript preparation

_X_No ___Yes, money paid to you ___Yes, money paid to institution* Name of entity___ Comments___

8. Patents (planned, pending or issued)

_X_No ___Yes, money paid to you ___Yes, money paid to institution* Name of entity___ Comments___

9. Royalties

_X_No ___Yes, money paid to you ___Yes, money paid to institution* Name of entity___ Comments___

10. Payment for development of educational presentations

_X_No ___Yes, money paid to you ___Yes, money paid to institution* Name of entity___ Comments___

11. Stock/stock options

_X_No ___Yes, money paid to you ___Yes, money paid to institution* Name of entity___ Comments___

12. Travel/accommodations/ meeting expenses unrelated to activities listed**

_X_No ___Yes, money paid to you ___Yes, money paid to institution* Name of entity___ Comments___

13. Other (err on the side of full disclosure)

_X_No ___Yes, money paid to you ___Yes, money paid to institution* Name of entity___ Comments___

** For example, if you report a consultancy above there is no need to report travel related to that consultancy on this line.

Section 4. Other relationships

Are there other relationships or activities that readers could perceive to have influenced, or that give the appearance of potentially influencing, what you wrote in the submitted work?

_X_No other relationships/conditions/circumstances that present a potential conflict of interest

__Yes, the following relationships/conditions/circumstances are present (explain below):

>>>>>>>>>>><<<<<<<<<<<

The International Committee of Medical Journal Editors

The ICMJE Disclosure of Potential Conflicts of Interest Form was adopted by *Arthroscopy: The Journal of Arthroscopic and Related Surgery* along with 17 other leading orthopaedic journals at the 2011 annual meeting of the American Academy of Orthopaedic Surgeons.
